# Early versus standard initiation of renal replacement therapy in furosemide stress test non-responsive acute kidney injury patients (the FST trial)

**DOI:** 10.1186/s13054-018-2021-1

**Published:** 2018-04-19

**Authors:** Nuttha Lumlertgul, Sadudee Peerapornratana, Thananda Trakarnvanich, Wanjak Pongsittisak, Kajbundit Surasit, Anan Chuasuwan, Pleumjit Tankee, Khajohn Tiranathanagul, Kearkiat Praditpornsilpa, Kriang Tungsanga, Somchai Eiam-Ong, John A. Kellum, Nattachai Srisawat, for the FST Study Group

**Affiliations:** 1 Division of Nephrology, Department of Medicine, Faculty of Medicine, Chulalongkorn University, Bangkok, Thailand; 2 Excellence center for Critical Care Nephrology, King Chulalongkorn Memorial Hospital, Thai Red Cross Society, Bangkok, Thailand; 3 Renal Division, Department of Medicine, Vajira Hospital, Navamindradhiraj University, Bangkok, Thailand; 4 Nakhon Ping Hospital, Chiang Mai, Thailand; 5 Bhumibol Adulyadej Hospital, Bangkok, Thailand; 6 Vajira Phuket Hospital, Phuket, Thailand; 7 The Center for Critical Care Nephrology, CRISMA, Department of Critical Care Medicine, University of Pittsburgh School of Medicine, Pittsburgh, PA USA

**Keywords:** Furosemide stress test, Acute kidney injury, Renal replacement therapy

## Abstract

**Background:**

The timing of initiation of renal replacement therapy (RRT) in severe acute kidney injury (AKI) remains controversial, with early initiation resulting in unnecessary therapy for some patients while expectant therapy may delay RRT for other patients. The furosemide stress test (FST) has been shown to predict the need for RRT and therefore could be used to exclude low-risk patients from enrollment in trials of RRT timing. We conducted this multicenter pilot study to determine whether FST could be used to screen patients at high risk for RRT and to determine the feasibility of incorporating FST into a trial of early initiation of RRT.

**Methods:**

FST was performed using intravenous furosemide (1 mg/kg in furosemide-naive patients or 1.5 mg/kg in previous furosemide users). FST-nonresponsive patients (urine output less than 200 mL in 2 h) were then randomized to early (initiation within 6 h) or standard (initiation by urgent indication) RRT.

**Results:**

FST was completed in all patients (100%). Only 6/44 (13.6%) FST-responsive patients ultimately received RRT while 47/60 (78.3%) nonresponders randomized to standard RRT either received RRT or died (*P* <  0.001). Among 118 FST-nonresponsive patients, 98.3% in the early RRT arm and 75% in the standard RRT arm received RRT. The adherence to the protocol was 94.8% and 100% in the early and standard RRT group, respectively. We observed no differences in 28-day mortality (62.1 versus 58.3%, *P* = 0.68), 7-day fluid balance, or RRT dependence at day 28. However, hypophosphatemia occurred more frequently in the early RRT arm (*P* = 0.002).

**Conclusion:**

The furosemide stress test appears to be feasible and effective in identifying patients for randomization to different RRT initiation times. Our findings should guide implementation of large-scale randomized controlled trials for the timing of RRT initiation.

**Trial registration:**

clinicaltrials.gov, NCT02730117. Registered 6 April 2016.

**Electronic supplementary material:**

The online version of this article (10.1186/s13054-018-2021-1) contains supplementary material, which is available to authorized users.

## Background

Acute kidney injury (AKI) is one of the most common and serious complications in critical care patients [1–3]. Renal replacement therapy (RRT) provides cornerstone management in severe AKI. While RRT is initiated promptly for life-threatening indications (e.g., severe hyperkalemia), there is controversy as to whether earlier initiation is beneficial in the absence of urgent indications [4–6]. Two recent randomized trials reached opposite conclusions as to whether early initiation is beneficial [7, 8]. The Artificial Kidney Initiation in Kidney Injury (AKIKI) trial used AKI stage 3 and conventional indications for RRT initiation. Early initiation was not superior to a conservative approach, and the lowest mortality was observed in patients who never received RRT. In the standard RRT group, 49% of the patients spontaneously recovered [8]. In the Effect of Early vs Delayed Initiation of Renal Replacement Therapy on Mortality in Critically Ill Patients with Acute Kidney Injury (ELAIN) study, however, AKI stage 2 plus plasma neutrophil gelatinase-associated lipocalin (NGAL) more than 150 ng/mL were used as inclusion criteria. Patients in the standard group had a 90% chance of RRT. This shows that using a biomarker could be an important prognostic criterion for the prediction of RRT requirement.

Unfortunately, it is difficult to predict the requirement for RRT, and clinicians vary widely in their decision making regarding when to initiate therapy. Recently, the furosemide stress test (FST) has been validated in patients with AKI stage 1 and 2 by the Kidney Disease Improving Global Outcomes (KDIGO) criteria as a novel test for the prediction of progression to AKI stage 3, the need for RRT, and in-hospital death [9]. FST also outperformed several novel biomarkers for the prediction of adverse outcomes [10]. Therefore, FST might be suitable to risk stratify AKI patients in guiding the decision to initiate RRT.

However, before embarking on a large trial to test alternate strategies for RRT initiation, we sought to determine whether FST could be used in a clinical trial setting to stratify AKI patients and to determine the feasibility of using FST in this setting. We therefore conducted a pilot study comparing early or standard initiation of RRT in FST-nonresponsive AKI patients. We also examined 28-day mortality and other clinical outcomes, although we did not power our study for these endpoints.

## Methods

### Trial design and oversight

The FST trial was funded by the Kidney Foundation of Thailand. The study was a prospective, multicenter, open-label, two-group randomized trial conducted in five intensive care units (ICU) in Thailand from March 2016 to July 2017. The trial was registered at clinicaltrials.gov (NCT02730117). The institutional ethics boards of all participating centers approved the protocol. The investigators informed patients or their surrogates about the trial both orally and with a written document. Informed consent was obtained from participating patients or their substitute decision-makers before the FST was performed. Co-investigators at each participating site were responsible for enrolling patients, ensuring adherence to the protocol, and completing the case record form. All analyses were performed by an independent statistician in accordance with the International Conference on Harmonization Good Clinical Practice Guidelines.

### Patients

All adult patients (≥ 18 years old) admitted to the ICU were screened. Patients with AKI at any stage (defined by KDIGO criteria) [11] were assessed for additional inclusion criteria (both of the following needed to be fulfilled): 1) clinical diagnosis of acute tubular necrosis (e.g., the presence of granular or epithelial cast, fractional excretion of sodium ≥ 1%, fractional excretion of urea ≥ 50%, plasma NGAL ≥ 150 ng/mL); 2) in the opinion of the treating team, the patient was well resuscitated and euvolemic; and 3) in the opinion of the treating team, the patient had neither an emergent indication nor a contraindication to RRT (Additional file 1: Appendix 1). We excluded patients with any of the following criteria: 1) baseline serum creatinine ≥ 2 mg/dL (male) or ≥ 1.5 mg/dL (female) [12]; 2) history of renal allograft; 3) known pregnancy; 4) allergy or known sensitivity to loop diuretics; 5) moribund patients with expected death within 24 h or whose survival to 28 days was unlikely due to an uncontrollable comorbidity (i.e., end-stage liver or heart disease, untreatable malignancy); 6) patients with advanced directives who issued the desire not to be resuscitated; 7) prior treatment with RRT within 30 days; 8) serum albumin < 2 g/dL; and 9) patients receiving extracorporeal membrane oxygenation or circulatory assistance (Additional file 1: Appendix 1). We considered the patients to be provisionally eligible if all the inclusion criteria were met, and no exclusion criteria were present.

### Furosemide stress test (FST)

FST was performed by giving intravenous furosemide 1 mg/kg to naive patients or 1.5 mg/kg to patients with a history of furosemide use within 7 days. Urine output was measured hourly and, if the urine output exceeded 200 mL for the subsequent 2 h, the patient was considered to be FST responsive. Patients with a urine output less than 200 mL in 2 h were considered FST nonresponsive and underwent randomization. Additional data are provided in Additional file 1: Table S1 [9].

### Randomization

We randomized patients 1:1 to early or standard RRT initiation using a randomly permuted block of four, stratified by center and type of ICU. Patients randomized to early RRT were to receive RRT within 6 h of randomization [7]. The 6-h period was for the establishment of vascular access and RRT initiation. In the standard RRT group, RRT was initiated only if one of the following criteria were met: blood urea nitrogen ≥ 100 mg/dL, serum potassium > 6 mmol/L, serum bicarbonate < 12 mmol/L or pH < 7.15, PaO_2_/FiO_2_ ratio < 200, or chest radiograph compatible with pulmonary edema.

### Interventions

The starting RRT modality was continuous venovenous hemofiltration (CVVH) using integrated machines (The Prismaflex® system, Gambro, Sweden or the Aquarius™ system, Nikkiso, Japan) with high-flux hemofilters (AN69 or HF12) and pre-filter replacement fluid of 25–30 mL/kg/h. The blood flow target was 150–200 mL/min. Regional citrate anticoagulant was the first-line of the anticoagulation strategy, followed by heparin in patients requiring systemic anticoagulation, and no anticoagulation in patients with contraindications to citrate or coagulopathy. Once the patients became hemodynamically stable (a decreasing dose or no longer needing the use of inotropic drugs) or were transferred out of the ICU, the RRT modality could be switched to prolonged intermittent RRT (PIRRT) or intermittent hemodialysis according to the judgment of the treating physician. RRT was continued until death, patient withdrawal, or renal recovery. The anticoagulant of choice in our study was intravenous heparin. However, if the patients had a contraindication to heparin, we used a no anticoagulant strategy. Renal recovery was defined as spontaneous diuresis more than 1000 mL/day or 2000 mL/day with diuretics with resolution of electrolyte or acid-base abnormalities and did no requirement to resume RRT for at least 7 days [8].

### Follow-up and data collection

We followed all patients for 28 days from randomization or until hospital discharge and serially assessed the severity of illness, laboratory data, and physiological data. RRT use and prescription details were recorded. We collected blood samples at days 0, 3, and 7 to measure: serum N-terminal prohormone of brain natriuretic peptide (NT-proBNP), a marker of volume overload [13]; plasma NGAL, a marker of kidney injury [14]; and serum angiopoietin-2, a marker of endothelial dysfunction [15].

### Outcomes

The primary outcome was feasibility as judged by: 1) compliance with the study protocol for > 90% of patients; 2) the ability to use FST to differentiate the RRT rate in FST responders and standard group of nonresponders 50%; 3) successful randomization of FST nonresponders; 4) separation of timing of RRT initiation between the early and standard RRT groups for at least 24 h; and 5) < 10% lost to follow-up.

Secondary outcomes included: 1) 28-day all-cause mortality; 2) 7-day fluid balance; 3) ICU-free days; 4) mechanical ventilator-free days; 5) RRT-free days; 6) length of ICU stay and hospital stay; 7) renal recovery; 8) dialysis requirement on day 28; 9) the proportion of patients free from RRT on days 0, 3, and 7; 10) nonrenal Sequential Organ Failure Assessment (SOFA) score on days 0, 3, and 7; and 11) RRT-related adverse events and vascular access-related adverse events (Additional file 1: Appendix 2). Exploratory endpoints included biomarkers (plasma NGAL and angiopoietin-2, and serum NT-proBNP) on days 0, 3, and 7.

### Biomarker assays

Blood samples were drawn in pyrogen-free vials and plasma was separated by centrifugation and frozen (−80 °C). Blood samples for NGAL, angiopoietin-2, and NT-proBNP were determined at trial inclusion, and day 3 and day 7 after randomization. Plasma biomarkers (NGAL and angiopoietin-2) were tested using enzyme-linked immunosorbent assay (R&D Systems, USA). Serum NT-proBNP was determined using electrochemiluminescence immunoassay analysis (Cobas assay; Roche Diagnostics, Mannheim, Germany).

### Sample size determination

To detect a 10.0% difference in 28-day mortality rate between early and standard RRT with a power of 80% and a 5% significance level on the basis of a previous report, approximately 900 patients would be needed [7]. As a feasibility study, we aim to use FST to risk stratify patients who would need RRT and not need RRT. At least thirty patients were required to detect a 50% absolute difference in the proportion of RRT between FST responders and FST nonresponders (standard group) with a power of 80% (β = 0.2) at a 5% significance level (α = 0.05). The trial was terminated on 31 July 2017 after recruiting 162 patients (44 FST responders and 118 FST nonresponders).

### Statistical analysis

All analyses adhered to the intention-to-treat principle. Categorical data are described as numbers and percentages and compared between treatment groups using Chi-square or Fisher’s exact test. Continuous variables are described as means (with standard deviations (SD)) or medians (with interquartile range (IQR)) and compared between each group using unpaired *t* test in normally distributed data or Wilcoxon rank sum test for non-normal data. Overall survival for all patients was estimated by the Kaplan-Meier method. A log-rank test was used to compare time to death between treatment arms and secondarily among patients undergoing RRT versus no RRT, and for patients with positive versus negative FST. The univariate Cox proportional hazard regression model was used to determine factors associated with RRT requirement in the standard arms using *p* values < 0.10, and a multivariate model was analyzed using significant factors from the univariate model, including gender. Data from all the patients were censored at the time of death or at day 28. Severity score, laboratory data, and physiological data between days 0, 3, and 7 were computed using repeated measures analysis of variance (ANOVA) for differences within groups and generalized estimating equations for differences between groups. All analyses were performed using Stata 14.0.

## Results

### Cohort characteristics and feasibility outcomes

**Fig. 1:**
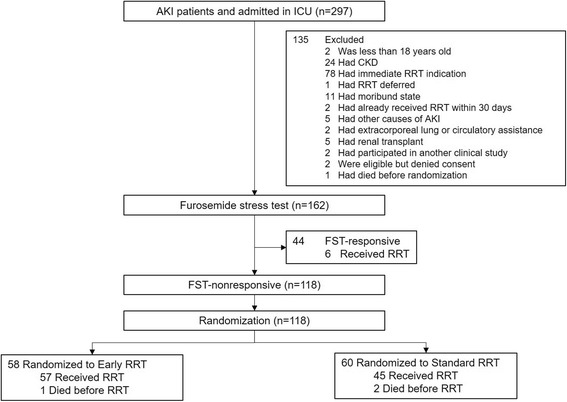
Flowchart of patient allocation. AKI, acute kidney injury; CKD, chronic kidney disease; FST, furosemide stress test; ICU, intensive care unit; RRT renal replacement therapy

Among 297 patients with AKI potentially eligible for inclusion in this trial, 162 patients underwent FST (Fig. 1). Forty-four patients were FST responsive, while 118 patients were FST nonresponsive and were randomized to early RRT (*n* = 58) or standard RRT (*n* = 60). Compliance with the study protocol for all patients is shown in Table 1. Sites were able to perform FST in all eligible patients. The FST successfully excluded patients at low risk for RRT: 6/44 (13.6%) of FST-responsive patients subsequently underwent RRT. Conversely, among FST-nonresponsive patients randomized to standard RRT, 45/60 (75%) underwent RRT (*P* <  0.001). A comparison of baseline characteristics between FST-responsive and -nonresponsive patients is provided in Additional file 1: Table S1. Significantly fewer FST-responsive patients were on vasopressors (*P* = 0.016), had lower severity scores (Acute Physiology and Chronic Health Evaluation (APACHE) II and SOFA score; *P* < 0.001), and less severe AKI (*P* = 0.001) compared with FST-nonresponsive patients. Sepsis was present in 52.3% of FST-responsive patients compared with 58.5% of FST-nonresponsive patients. Of the 44 FST-responsive patients, 34.1% died, whereas 60.2% of the FST-nonresponsive patients died (*P* = 0.003). When visualized by receiver operating characteristics, FST had a higher area under the curve (AUC) (0.83) than APACHE II (0.71), SOFA (0.75), and nonrenal SOFA score (0.72) for the prediction of RRT (Additional file 1: Table S2).

**Table 1:** Study protocol compliance

Parameters	FST nonresponsive (*n* = 118)		FST responsive (*n* = 44)
	Early RRT (*n* = 58)	Standard RRT (*n* = 60)	
FST completion, *n* (%)	58 (100)	60 (100)	44 (100)
RRT, *n* (%)	57 (98.3)	45 (75)	6 (13.6)
Initiation of RRT within 6 h of randomization, *n* (%)	49/58 (84.5%)^a^	N/A	N/A
Initiation of RRT within 12 h of randomization, *n* (%)^b^	55/58 (94.8)	N/A	N/A
Adherence to standard RRT initiation, *n* (%)	N/A	45/45 (100)	6/6 (100)
Death after meeting RRT criteria but prior to RRT initiation, *n* (%)	1 (1.7)	2 (3.3)	0 (0)
Loss to follow-up, *n* (%)	0 (0)	0 (0)	0 (0)

FST, furosemide stress test; N/A, not applicable; RRT, renal replacement therapy

^a^Early RRT = RRT initiation within 6 h after randomization; standard RRT = RRT initiation according to standard indications

^b^One patient died before RRT initiation. Two patients received RRT but later than 12 h due to the necessity for intervention

Randomization appeared to be successful since baseline characteristics were well balanced between treatment arms, except for APACHE II score (Table 2). The early RRT group had a significantly higher APACHE II score compared with the standard RRT group (24.5 versus 21.8, *P* = 0.027). Most patients had AKI stage 2 and 3 (80.0%). Sepsis was present in 58.6% (Table 2).

**Table 2:** Demographic, clinical, and biochemical data between early RRT and standard RRT patients

Parameters	Early RRT (*n* = 58)	Standard RRT (*n* = 60)	*P* value
Age (years), mean (SD)	67.5 (15.0)	66.7 (16.7)	0.80
Male, *n* (%)	29 (50)	29 (48.3)	0.86
ICU, *n* (%)			0.79
Medical	40 (69)	40 (66.7)	
Surgical	18 (31)	20 (33.3)	
Mechanical ventilation, *n* (%)	48 (82.8)	50 (83.3)	0.93
Vasopressors, *n* (%)	45 (77.6)	47 (78.3)	0.92
Sepsis, *n* (%)	37 (63.8)	32 (53.3)	0.25
APACHE II score, mean (SD)	24.5 (6.4)	21.8 (6.9)	**0.027**
SOFA score, mean (SD)	12.7 (3.3)	11.4 (4.0)	0.058
Nonrenal SOFA score, mean (SD)	9.9 (3.3)	9.1 (4.1)	0.21
Baseline serum creatinine (mg/dL), mean (SD)	1.14 (0.44)	1.03 (0.37)	0.16
Estimated GFR (mL/min/1.73m^2^), mean (SD)^a^	70.31 (28.1)	69.98 (22.8)	0.95
AKI staging, *n* (%)			0.06
1	11 (19)	12 (20)	
2	27 (46.6)	16 (26.7)	
3	20 (34.5)	32 (53.3)	
Blood urea nitrogen at enrollment (mg/dL), median (IQR)	42 (37–78)	51 (37.5–61.25)	0.52
Serum creatinine at enrollment (mg/dL), median (IQR)	2 (2–3)	2.5 (2–3)	0.88
Co-morbidities, *n* (%)			
Hypertension	29 (50)	24 (56.7)	0.47
Diabetes	14 (24.1)	15 (25)	0.91
Dyslipidemia	16 (27.6)	16 (26.7)	0.91
Ischemic heart disease	12 (20.7)	10 (16.7)	0.58
Malignancy	12 (20.7)	8 (13.3)	0.29
Cerebrovascular disease	5 (8.6)	7 (11.7)	0.58
Chronic liver disease	10 (17.2)	11 (18.3)	0.88
Nephrotoxic drugs, n (%)			
Colistin	5 (8.6)	10 (16.7)	0.19
Vancomycin	1 (1.7)	1 (1.7)	0.98
Contrast	8 (13.8)	11 (18.3)	0.50
Aminoglycosides	2 (3.4)	2 (3.3)	0.97
Amphotericin	2 (3.4)	0 (0)	0.15
NSAIDs	2 (3.4)	1 (1.7)	0.54
Cardiac surgery, *n* (%)	13 (22.4)	8 (13.3)	0.20
Treatment limitation, *n* (%)^b^	12 (20.7)	10 (16.7)	0.58
Fluid accumulation at randomization (mL), median (IQR)	4763 (2837–8515)	5114 (2050–8803)	0.84
Percentage of fluid overload, median (IQR)^c^	9.53 (3.43–19.68)	7.63 (2.10–12.02)	0.87
Baseline NGAL (ng/mL), median (IQR)	625 (376–1362)	860 (447–1204)	0.63
Baseline NT-proBNP (pg/mL), median (IQR)	4301 (515–35,000)	5844 (869–10,007)	0.71
Baseline angiopoietin-2 (ng/mL), median (IQR)	16,784 (8649–35,545)	22,294 (12539–33,186)	0.95

Significant values are shown in bold type

AKI, acute kidney injury; APACHE II, Acute Physiology and Chronic Health Evaluation; GFR, glomerular filtration rate; ICU, intensive care unit; IQR, interquartile range; NGAL, neutrophil gelatinase-associated lipocalin; NSAID, nonsteroidal anti-inflammatory drug; NT-proBNP, N-terminal prohormone of brain natriuretic peptide; RRT, renal replacement therapy; SD, standard deviation; SOFA, Sequential Organ Failure Assessment

^a^Estimated GFR was calculated by the CKD-EPI creatinine equation (2009)

^b^Treatment limitation is defined as withholding or withdrawal of patients from the treatment of primary disease either by the surrogates’ decision or after a period of intensive care management

^c^Fluid overload is calculated by the total volume of fluid accumulation (intake – output) since ICU admission divided by body weight on admission and reported as a percentage

Median time from randomization to RRT initiation was 2 (IQR 1–3) h in the early RRT group and 21 (IQR 17–49) h in the standard RRT group (difference = 19 h; *P* < 0.001). The median time from ICU admission to RRT initiation and median time from oliguria to RRT initiation in the early and standard RRT groups was 22 versus 100 h and 17 versus 38 h (*P* < 0.001) in both groups (Table 3). No patients were lost to follow-up for the survival status at day 28.

**Table 3:** Duration parameters in the intervention trial

Parameters	Early RRT (*n* = 58)	Standard RRT (*n* = 60)	*P* value
Time from randomization to RRT (h), median (IQR)	2 (1–3)	21 (16.75–48.5)	< 0.001
Time from ICU admission to RRT (h), median (IQR)	22 (14–51)	100 (25–257)	< 0.001
Time from oliguria to RRT (h), median (IQR)	17 (11–24)	37.5 (30–55)	< 0.001
Fluid accumulation from randomization to RRT (mL), median (IQR)	4763 (2837–8515)	8659 (4388–10,465)	0.02

ICU, intensive care unit; IQR, interquartile range; RRT, renal replacement therapy

In the early RRT arm, 57 out of 58 patients received RRT as 1 patient died before RRT initiation. In the standard RRT group, 45 out of 60 (75.0%) eventually met the prespecified indications and received RRT and 2 died prior to RRT. Interestingly, 15 out of 60 (25%) showed spontaneous renal recovery (Fig. 1). In the standard arm, multivariate Cox proportional hazard regression analysis showed that SOFA score, sepsis, and baseline plasma NGAL were significant predictors for RRT requirement. Patients who spontaneously recovered had median baseline plasma NGAL level of 518.5 (IQR 397.5–641.5) ng/mL compared with 885.5 (IQR 450–1320) ng/mL in those who eventually required RRT. Plasma NGAL had an adjusted hazard ratio (HR) of 1.06 (95% confidence interval (CI) 1.01–1.12; *P* = 0.024) for RRT requirement.

Cumulative fluid balance from ICU admission to randomization was comparable between both groups (4763 (IQR 2837–8515) mL in the early group versus 5114 (IQR 2050–8803) mL in the standard group). RRT prescription including CVVH dose and median ultrafiltration rate per day did not differ between both groups.

### Secondary outcomes

**Fig. 2:**
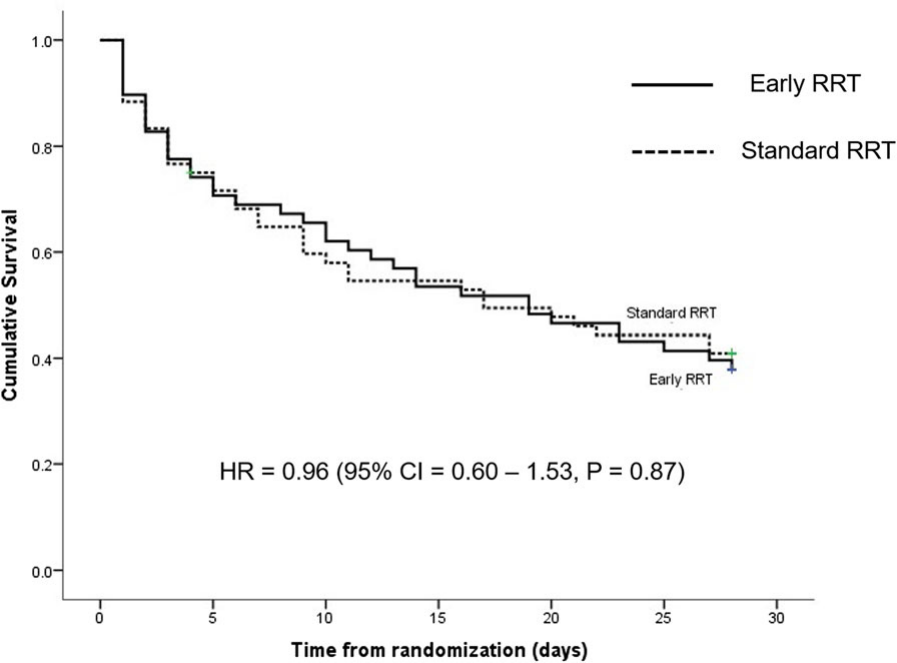
Survival curves of patients receiving early and standard renal replacement therapy (RRT) (straight line, early RRT group; dashed line, standard RRT group). The figure shows the Kaplan-Meier curve of the probability of survival from randomization to day 28. CI, confidence interval; HR, hazard ratio

Mortality rates were estimated by the Kaplan-Meier method. The overall mortality at day 28 was 60.2%. The 28-day mortality rate in the early RRT group did not differ from the standard RRT group (62.1% versus 58.3%, *P* = 0.68; unadjusted HR 0.96 (95% CI 0.60–1.53), *P* = 0.87) (Fig. 2). Adjusted HR for APACHE II was 1.06 (95% CI 0.66–1.69; *P* = 0.81). The mortality rate between RRT and no RRT in the standard RRT group was also not different (HR for RRT versus no RRT 1.59 (95% CI 0.85–4.97), *P* = 0.11) (Additional file 1: Figure S1).

There were no significant differences in renal recovery, cumulative fluid balance on the first 7 days, RRT-free days, mechanical ventilation-free days, ICU-free days, or dialysis dependence on day 28 between the two groups (Table 4). The levels of plasma NGAL, NT-proBNP, and angiopoietin-2 at the time of randomization were high. There were no significant differences in these three biomarkers on days 0, 3, and 7 within treatment arm and between treatment arms (Additional file 1: Table S3).

**Table 4:** Outcomes in the intervention trial

Outcomes	Early RRT (*n* = 58)	Standard RRT (*n* = 60)	*p* value
Primary outcome			
Mortality, *n* (%)	36 (62.1)	35 (58.3)	0.68
Secondary outcomes			
Recovery, *n* (%)	21 (36.2)	19 (31.7)	0.60
7-day fluid balance (mL), median (IQR)	−1702 (−5610 to 2129)	−1247 (−4535 to 1581)	0.75
Mean RRT dose (mL/kg/h), mean (SD)	26.8 (5.3)	26.3 (8.9)	0.73
RRT-free days, median (IQR)	0 (0–19)	0 (0–28)	0.64
MV-free days, median (IQR)	4 (0–24)	0.5 (0–20.3)	0.66
ICU-free days, median (IQR)	14 (0–21)	4.5 (0–18)	0.46
ICU length of stay (days), median (IQR)	12 (7–26)	13.5 (9–29)	0.76
Hospital length of stay (days), median (IQR)	26 (19–53)	28.5 (17–55.3)	0.82
Renal replacement therapy dependency at day 28, *n* (%)	7 (12.1)	10 (16.7)	0.77

ICU, intensive care unit; IQR, interquartile range; MV, mechanical ventilation; RRT, renal replacement therapy

### Adverse effects

RRT-related and central venous catheter (CVC)-related adverse events are shown in Additional file 1: Table S4. There was significantly more hypophosphatemia in the early RRT group (*P* = 0.002). There were more CVC-related malfunctions and an incidence of air embolism in the early RRT group (*P* = 0.038). Other RRT-related and CVC-related adverse events were comparable (Additional file 1: Table S4).

## Discussion

In this pilot randomized controlled trial (RCT), we demonstrated the feasibility and safety of conducting a trial comparing early versus standard RRT using FST as an initial triage strategy. The results of the present study demonstrate that FST was easy to administer in the context of a clinical trial (100% compliance) and provided excellent predictive ability for the subsequent use of RRT; nonresponsive patients had an RRT rate of 75% versus 13.6% for FST-responsive patients (Additional file 1: Table S1). Compliance with other aspects of the study protocol was also excellent, with > 95% of patients receiving the intervention they were randomized to receive. Randomization was successful in that few baseline differences were seen between intervention arms and the separation of timing of initiation (early versus standard) approached, but did not quite achieve, 24 h. Finally, we achieved excellent follow-up, with 100% of patients available for survival analysis.

We did not encounter any safety issues using the FST, and the only adverse events encountered with early initiation were increased rates of hypophosphatemia and dialysis catheter issues (Additional file 1: Table S4). Our sample may have been too small to detect some adverse events. For example, there was more hemodynamic instability (34.5% versus 20%) with early initiation while less cumulative fluid removal was seen at 7 days with standard RRT (1.2 versus 1.7 L), but neither of these differences were significant. However, there was a significant difference in fluid accumulation from randomization to initiation of RRT (4.8 versus 8.7 L, *P* = 0.02) favoring early initiation.

Our final sample size was insufficient to test whether timing of initiation of RRT impacted 28-day survival. However, we did not observe any differences in survival between early and standard RRT (Table 4, Fig. 2). Other secondary outcomes including renal recovery rate, ICU-free days, mechanical ventilator-free days, 7-day fluid balance, and dialysis dependence rate were not significantly different between both treatment arms (Table 4), although we were unpowered for many of these endpoints. For example, ventilator-free days and ICU-free days were both greater with early initiation but with very wide confidence intervals. Similarly, the small differences observed in ICU and hospital length of stay (difference of 1.5 and 2.5 days, respectively) are clinical relevant but would have required a much larger trial to detect.

The optimal timing to initiate RRT in AKI patients remains to be established [7, 8, 16–25]. Two recently published RCTs examining timing of RRT initiation reached different conclusions. The AKIKI multicenter trial in France investigated early initiation (within 6 h after documentation of KDIGO stage 3) versus a “wait and see” strategy (as per conventional indications). Sepsis, severe sepsis, or septic shock were present in 80%. Mortality at 60 days was not different between the two strategies [8]. Conversely, a single center RCT in Germany (ELAIN study) defined early RRT as AKI KDIGO stage 2 plus plasma NGAL > 150 ng/mL and delayed RRT as AKI stage 3. Early initiation of RRT significantly reduced 90-day mortality compared with delayed initiation (39.3% versus 54.7%) [7]. The other ongoing study, Standard versus Accelerated initiation of Renal Replacement Therapy in Acute Kidney Injury (STARRT-AKI), also uses the higher cut-off level of plasma NGAL (≥ 400 ng/mL) as one of the three inclusion criteria along with a twofold rise in serum creatinine and oliguria [26]. By using plasma NGAL as a screening biomarker to filter patients, the ELAIN trial was able to select 90% of patients in the standard arm who required RRT. On the contrary, 49% of the patients in the standard indication arm of the AKIKI trial, which used only AKI staging as a screening tool, showed spontaneous recovery, which implied that RRT could also be avoided in some patients in the early indication arm had there been screening tools for selection of high-risk patients. Therefore, a pure clinical strategy may not be enough to analyze early versus standard initiation strategy and prevent unnecessary RRT. In our study, we identified 44/162 (27.2%) FST-responsive patients with only a 13.6% rate of RRT. RRT was averted in 86.4% of FST responders. We were able to select a group with a 75% RRT rate in the standard RRT group of FST nonresponders. In the standard arm, plasma NGAL was also a significant predictor for spontaneous recovery. This suggests that while FST is an excellent strategy to select patients who would recover, combining FST nonresponsiveness with plasma NGAL might be an even more suitable strategy to predict patients who are likely to require RRT.

There are some limitations in our study. First, due to the nature of the study, this was an unblinded RCT. The robust protocol for initiation of RRT and high compliance rates minimizes the risk of bias in RRT initiation. Second, the numbers of participants were rather small (60 in each arm) leading to insufficient power for secondary endpoints. However, as a pilot study, our results support the feasibility and safety of this approach for a definitive trial in the future. We were likely underpowered for our exploratory biochemical analysis as well. The incidence of hypophosphatemia was higher in the early RRT group, and severe hypophosphatemia is known to be associated with respiratory failure and weaning failure [27]. Plasma NGAL, NT-proBNP, and angiopoietin-2 levels in the early intervention arm were not significantly different from standard RRT. However, there were wide confidence intervals and important differences could have been missed.

## Conclusion

This is the first pilot study to demonstrate the use of FST to identify patients with high risk of AKI progression and to investigate whether early RRT could improve clinical outcomes in this subgroup of patients. FST had excellent predictive ability for the subsequent use of RRT. Larger trials powered for clinical outcomes should be enabled by our results.

## Additional file

Additional file 1: **Table S1.** Demographic, clinical, and biochemical data between FST-nonresponsive and FST-responsive patients. **Table S2.** Multivariable logistic regression on parameters to predict RRT. **Table S3.** Comparison of severity score and plasma biomarkers in the intervention trial. **Table S4.** Adverse events in the intervention trial. **Appendix 1.** Inclusion and exclusion criteria for patients in the FST study. **Appendix 2.** Definitions of safety outcomes related to the administration of RRT or vascular access for RRT. **Figure S1.** Survival curves of patients in the standard RRT arm who received and did not receive RRT (blue line, no RRT group; red line, RRT group). The figure shows the Kaplan-Meier curve of the probability of survival from randomization to day 28. (DOCX 520 kb)

## Abbreviations

AKI: Acute kidney injury
AKIKI: Artificial Kidney Initiation in Kidney Injury
APACHE II: Acute Physiology and Chronic Health Evaluation
AUC: Area under the curve
CI: Confidence interval
CVC: Central venous catheter
CVVH: Continuous venovenous hemofiltration
ELAIN: Effect of Early vs Delayed Initiation of Renal Replacement Therapy on Mortality in Critically Ill Patients with Acute Kidney Injury
FST: Furosemide stress test
HR: Hazard ratio
ICU: Intensive care unit
IQR: Interquartile range
KDIGO: Kidney Disease Improving Global Outcomes
NGAL: Neutrophil gelatinase-associated lipocalin
NT-proBNP: N-terminal prohormone of brain natriuretic peptide
PIRRT: Prolonged intermittent renal replacement therapy
RCT: Randomized controlled trial
RRT: Renal replacement therapy
SD: Standard deviation
SOFA: Sequential Organ Failure Assessment
