# Supplementary material for: Early versus standard initiation of renal replacement therapy in furosemide stress test non-responsive acute kidney injury patients (the FST trial)
Source: Crit Care. 2018 Apr 19;22:101. doi: 10.1186/s13054-018-2021-1 (PMC5909278; doi:10.1186/s13054-018-2021-1)

**Additional File**

**Table S1.** Demographic, clinical, and biochemical data between FST- nonresponsive and FST-responsive patients

| **Parameters** | **FST-nonresponsive**  **(n = 118)** | **FST- responsive (n=44)** | ***p* value** |
| --- | --- | --- | --- |
| Age, years, mean (SD) | 67.1 (15.8) | 61.6 (16.7) | 0.055 |
| Male, n (%) | 58 (49.2) | 29 (65.9) | 0.057 |
| ICU, n (%)   - Medical - Surgical | 80 (67.8)  38 (32.2) | 22 (50)  22 (50) | **0.037** |
| Mechanical ventilation, n (%) | 98 (83.1) | 38 (86.4) | 0.61 |
| Vasopressors, n (%) | 92 (78) | 26 (59.1) | **0.016** |
| Sepsis, n (%) | 69 (58.5) | 23 (52.3) | 0.48 |
| APACHE II score, mean (SD) | 23.1 (6.7) | 19.0 (5.3) | **< 0.001** |
| SOFA score, mean (SD) | 12.0 (3.7) | 8.6 (3.6) | **< 0.001** |
| Non-renal SOFA score, mean (SD) | 9.5 (3.7) | 7.0 (3.5) | **< 0.001** |
| Baseline serum creatinine, mg/dL, mean (SD) | 1.08 (0.41) | 1.09 (0.36) | 0.93 |
| Estimated GFR, mL/min/1.73m^2^, mean (SD)* | 70.1 (25.5) | 73.3 (27.4) | 0.49 |
| AKI staging, n (%)  1  2  3 | 23 (19.5)  43 (36.4)  52 (44.1) | 21 (47.7)  12 (27.3)  11 (25) | **0.001** |
| Blood Urea Nitrogen at enrollment, mg/dL, median [IQR] | 47.5 [33.75-66.25] | 37 [29-49] | **0.02** |
| Serum creatinine at enrollment, mg/dL, median [IQR] | 2 [2-3] | 2 [2-3] | 0.43 |
| RRT, n (%) | 103 (87.3) | 6 (13.6) | **< 0.001** |
| Mortality, n (%) | 71 (60.2) | 15 (34.1) | **0.003** |

Data is reported by mean ± standard deviation unless indicated otherwise

*eGFR by CKD-EPI creatinine equation (2009)

ICU, intensive care unit; APACHE II, acute physiology and chronic health evaluation; SOFA, Sequential Organ Failure Assessment ; eGFR, estimated glomerular filtration rate; AKI, acute kidney injury; RRT, renal replacement therapy

**TableS 2. Multivariable logistic regression on parameters to predict RRT**

| **Parameters** | **AUC** | **95% CI** |
| --- | --- | --- |
| FST | 0.83 | 0.75 - 0.91 |
| APACHE II | 0.71 | 0.63 – 0.79 |
| SOFA | 0.75 | 0.67 – 0.84 |
| Non-renal SOFA | 0.72 | 0.63 – 0.80 |

FST, furosemide stress test; APACHE II, acute physiology and chronic health evaluation; SOFA, sequential organ failure assessment

**Table S3. Comparison of severity score and plasma biomarkers day in Intervention trial**

| **Group** | **Early RRT** | | | | **Standard RRT** | | |  | ***p* value+** |
| --- | --- | --- | --- | --- | --- | --- | --- | --- | --- |
| **Parameters** | **Day 0 (N=58)** | **Day 3 (N=47)** | **Day 7 (N=40)** | ***p* value** | **Day 0**  **(N=60)** | **Day 3 (N=46)** | **Day 7 (N=39)** | ***p* value** |  |
| Non-renal SOFA score, mean (SD) | 9.9 (3.3) | 9.6 (4.1) | 8.1 (4.4) | 0.02 | 9.1 (4.1) | 7.8 (4.5) | 6.9 (4.4) | 0.03 | 0.09 |
| NGAL,^++^ ng/mL, median [IQR] | 894 [410.5- 1456.8] | 969  [577-1827] | 1172.5 [424.7-2004] | 0.02 | 770  [439-1320] | 654  [364-1721] | 651  [256- 1248] | 0.61 | 0.28 |
| NT-proBNP,^+++^ , pg/mL, median [IQR] | 4699  [920-35000] | 2545.9  [487.1- 18484] | 2581  [581.3- 21308] | 0.60 | 4231  [1684.3-13196] | 4884 [2024.3- 20323] | 3070.2 [994.6-18325] | 0.46 | 0.48 |
| Ang2^++++^  , ng/mL, median [IQR] | 19077  [10528- 41479] | 13561  [8053- 33304] | 10653.5 [4936- 14133] | <0.001 | 22829  [12096- 34468] | 12920  [7822- 31458] | 10476 [5677.5- 22901] | 0.004 | 0.65 |
| Urine output, mL, median [IQR] | 407.5  [185-1123] | 129  [22-610] | 145  [10-1080] | 0.03 | 690  [247.5- 1120] | 534  [65-2210] | 1385  [243- 2100] | 0.25 | 0.039 |

+ Testing the difference of parameters between groups using generalized estimating equation (GEE)

++ NGAL, neutrophil gelatinase associated lipocalin

+++ NT-proBNP, N-terminal prohormone of brain natriuretic peptide

++++ Ang2, angiopoietin 2

**Table S4. Adverse events in Intervention trial**

| **Adverse events** | **Early RRT**  **(n=58)** | **Standard RRT**  **(n=60)** | ***p* value** |
| --- | --- | --- | --- |
| Hemodynamic instability, n (%) | 20 (34.5) | 12 (20) | 0.08 |
| Arrhythmia, n (%) | 21 (36.2) | 16 (26.7) | 0.26 |
| Seizure, n (%) | 0 (0) | 0 (0) | 1.00 |
| Hypokalemia, n (%) | 3 (5.2) | 1 (1.7) | 0.29 |
| Hypophosphatemia, n (%) | 13 (22.4) | 2 (3.3) | **0.002** |
| Hypocalcemia, n (%) | 4 (6.9) | 4 (6.7) | 0.96 |
| CVC hemorrhage, n (%) | 1 (1.7) | 3 (5) | 0.33 |
| CVC pneumothorax, n (%) | 0 (0) | 0 (0) | 1.00 |
| CVC bacteremia, n (%) | 2 (3.4) | 2 (3.3) | 0.97 |
| CVC thrombosis, n (%) | 1 (1.7) | 0 (0) | 0.31 |
| Arterial puncture, n (%) | 2 (3.4) | 3 (5) | 0.68 |
| CVC others, n (%) | 4 (6.9)* | 0 (0) | **0.038** |

*****Catheter malfunction 3 cases, air embolism 1 case

CVC, central venous catheter

**Appendix 1: Inclusion and exclusion criteria for patients in the FST study**

**Inclusion criteria**

1. All adult patients (≥ 18 years old) Patients with AKI at any stage (defined by Kidney Disease Improving Global Outcomes (KDIGO) criteria)
2. Clinical diagnosis of acute tubular necrosis (e.g. presence of granular or epithelial cast, fractional excretion of sodium ≥ 1%, fractional excretion of urea ≥ 50%, plasma neutrophil gelatinase-associated lipocalin (NGAL) ≥ 150 ng/mL, absence of obstruction, glomerular or vascular disease)
3. Opinion of the treating team that the patient was well-resuscitated and euvolemic (e.g. fluid accumulation ≥ 5%, central venous pressure ≥ 8 mmHg, pulse pressure variation < 13%, inferior vena cava collapsibility index < 50% in spontaneously breathing patients or distensibility index < 18% in mechanically ventilated patients)
4. Opinion of the treating team that the patient had neither an emergent indication nor a contraindication to RRT.

**Exclusion criteria**

1. Baseline serum creatinine ≥ 2 (male) or ≥ 1.5 mg/dL (female)
2. History of renal allograft
3. Known pregnancy
4. Allergy or known sensitivity to loop diuretics
5. Moribund patients with expected death within 24 hours or whose survival to 28 days was unlikely due to an uncontrollable comorbidity (i.e. end-stage liver or heart disease, untreatable malignancy)
6. Patients with advanced directives issued the desire not to be resuscitated; prior treatment with RRT within 30 days
7. Serum albumin < 2 g/dL
8. Patients receiving extracorporeal membrane oxygenation or circulatory assistance

**Appendix 2: Definitions of safety outcomes related to administration of RRT or vascular access for RRT**

**RRT-associated hemodynamic instability** defined as hypotension requiring one of: initiation of a vasopressor during RRT session or need to escalate dose of a vasopressor during the RRT session or premature discontinuation of RRT session due to blood pressure drop or any other intervention to stabilize blood pressure during the dialysis session

**Arrhythmia, or seizure on RRT** As noted in the medical chart

**Hypokalemia** If serum potassium was below 3.0 mEq/L at any time during the study period

**Hypophosphatemia** If serum phosphate was below 1.5 mg/dL at any time during the study period

**Hypocalcemia** If albumin-adjusted total calcium was below 8 mg/dL at any time during the study period

**Hemorrhage at site of central venous catheter (CVC) insertion** defined as bleeding at the puncture site requiring transfusion of ≥ 1 unit(s) of packed red blood cells within 12 hours following insertion and/or surgical intervention/repair

**Pneumothorax** (for catheters placed in the internal jugular or subclavian positions) defined as air in the pleural space on routine chest x-ray that is performed following CVC insertion; further qualified by requirement for chest tube placement

**CVC-associated bacteremia** defined as bloodstream infection in 2 blood culture sets (one drawn from dialysis catheter and the other from another site) with no proven alternative source for bloodstream infection as per ICU attending OR culture-positive recovery of the same organism from the dialysis CVC upon removal OR culture-positive from the dialysis catheter within 2 hours prior to positive hemoculture from peripheral sites

**Ultrasonographically confirmed thrombus attributed to CVC** defined as any confirmed occlusive or non-occlusive thrombus in the vein in which a CVC was placed (or remains in place) or in the venous system drained by the vein in which the CVC was placed; further qualified by presence or absence of pulmonary embolism

**Air embolism** or suspected air embolism as documented in the medical record

**Arterial puncture at CVC insertion** as reported in the documentation of the CVC placement

**CVC malfunction** defined as inability to deliver blood flow for renal replacement therapy from malposition, occlusions by intraluminal thrombus or extrinsic fibrin sheath that require exchange or removal of the catheter

**Figure S1** Survival curves of patients in the standard RRT arm who received and did not receive RRT (Blue line, No RRT group; red line, RRT group). The figure shows Kaplan-Meier curve of the probability of survival from randomization to day 28


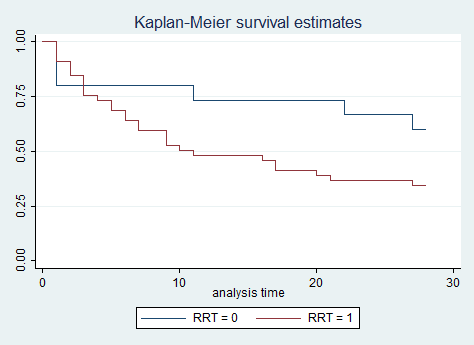

Supplement: Supplementary file 1 — Table S1. Demographic, clinical, and biochemical data between FST-nonresponsive and FST-responsive patients. Table S2. Multivariable logistic regression on parameters to predict RRT. Table S3. Comparison of severity score and plasma biomarkers in the intervention trial. Table S4. Adverse events in the intervention trial. Appendix 1. Inclusion and exclusion criteria for patients in the FST study. Appendix 2. Definitions of safety outcomes related to the administration of RRT or vascular access for RRT. Figure S1. Survival curves of patients in the standard RRT arm who received and did not receive RRT (blue line, no RRT group; red line, RRT group). The figure shows the Kaplan-Meier curve of the probability of survival from randomization to day 28. (DOCX 520 kb) [file 13054_2018_2021_MOESM1_ESM.docx]
